# Supplementary material for: Antimicrobial Dispensing Practices during COVID-19 and the Implications for Pakistan
Source: Antibiotics (Basel). 2023 Jun 6;12(6):1018. doi: 10.3390/antibiotics12061018 (PMC10294926; doi:10.3390/antibiotics12061018)
Supplement: Supplementary file 1 [file antibiotics-12-01018-s001.zip › antibiotics-2390479-supplementary.pdf]

Supplementary Material

Table S1. Initiatives to improve antibiotic utilization among physicians in ambulatory care in LMICs.

Country, Year and Reference	Intervention	Impact
Malaysia, 2006 [90]	Principally education involving academic detailing from the resident family medicine specialist alongside an information leaflet	• Reduction in general antibiotic prescribing rates from 14.3% of patients pre-intervention to 11.0% post-intervention (RR 0.77, 95% CI 0.72 to 0.83)<br>• Reduction in URTI-specific antibiotic prescribing rates from 27.7% and 16.6% post-intervention (RR 0.60, 95% CI 0.54 to 0.66)<br>• No significant change in prescribing habits among control physicians, i.e. those that did not participate in the study
Sudan, 2006 [91]	• 20 health centers in Khartoum State were randomly assigned to receive either (a) no intervention; (b) audit and feedback; (c) audit and feedback + seminar; or (d) audit and feedback + academic detailing<br>• The targeted interventions involved audit and feedback alongside academic detailing	• There was a significant reduction in the mean number of physician encounters where an antibiotic was prescribed by 6.3 and 7.7 ($p<0.001$) at 1 and 3 months post-intervention, respectively<br>• The mean number of encounters where antibiotics were inappropriately prescribed was also significantly reduced post intervention ($p<0.001$)
Bangladesh, 2007 [92]	• 3-arm study principally involving education divided into 3 groups:<br>• Group I – education surrounding current treatment guidelines and subsequent auditing of the prescribing of ARIs<br>• Group-II - Just education surrounding current treatment guidelines<br>• Group-III - Control group, i.e., no intervention)	• The prescribing of unnecessary antibiotics to treat ARIs was significantly reduced ($p<0.01$) compared to the pre-intervention period in Group I<br>• The average reduction in antibiotic prescribing (patient encounters) was 23.7% in Group I vs. 15.2% in Group II<br>• When factoring in the Control group (Group III), there was a 15.2% reduction in antibiotic prescribing in Group I and 6.9% in Group II
Nepal, 2009 [93]	Principally educational initiatives:<br>• Supervision/ monitoring via periodic visits by district supervisors to 41 primary health care (PHC) facilities<br>• Small-group training among prescribers followed by peer-group discussions as well as self-assessment of the data presented	• In children < 5 years, there was a significant improvement with respect to decreasing the prescribing of antimicrobials to treat children with diarrhea<br>• There was also a significant improvement in the prescribing of antibiotics for patients with URTIs without pneumonia
Thailand, 2014 [94]	Principally education which included:<br>• Training HCPs on the rational use of antibiotics<br>• Introduction of practice guidelines<br>• Potential for throat swabs (stool cultures for acute diarrhea)<br>• Printed brochures for patients/ relatives in waiting rooms. This included the potential harm associated with the overuse of antibiotics for URTIs and acute diarrhea	The multifaceted program resulted in:<br>• Limited prescribing of antibiotics for URTIs (13.0%) and for acute diarrhea (19.1%)<br>• Clinical responses on day 3 among patients after receiving care revealed more than 97% of patients who received antibiotics/ those who did not receive antibiotics were cured or improved

Kenya 2017 [95]	• A comprehensive set of interventions to improve the management of URTIs, UTIs, STIs and childhood diarrhea in PHCs.<br>• Interventions included:<br>o Online educational programs explaining the clinical guidelines for these 4 conditions<br>o 2-hour educational sessions<br>o Monthly feedback meetings<br>o Materials including posters and other material to remind prescribers of appropriate prescribing	• Adherence to agreed clinical quality measures (CQM) increased from 41.4% to 77.1% for PHCs that took part in the intervention but dropped slightly from 26.5% to 21.8% in controls over the 6-month study period. This was greatest for UTIs<br>• Adherence to CQMs significantly increased over the 6-month study period for the active intervention group<br>The interventions were well received by those operating in the PHCs
India, 2018 [96]	• Principally education to reduce the rate of inappropriate prescribing of antibiotics for URTIs<br>• Initiatives consisted of:<br>o Repeated process of audit and feedback combined with interactive training sessions<br>o One-to-one case-based discussions<br>o Guideline development and broadcasting alongside coding updates	• Antibiotic prescribing among 222 audited patients reduced from 62.6% of patients with URTIs prescribed antibiotics to 7.2% following the multiple interventions<br>• This was combined with an increase in the documentation of examination findings from 52.7% to 95.6% of patients justifying the treatment approach
Malaysia, 2019 [97]	Principally education involving educational toolkits included a training module for HCPs on URTI and acute diarrhea involving:<br>• One-hour educational sessions covering diagnostic criteria and treatment decision pathways<br>• Educational posters in Malay and English hung in patient waiting areas and consultation rooms<br>• Multimedia educational videos in the patient waiting areas<br>• Physician reminders	Appreciable reduction in antibiotic prescribing:<br>• URTIs down from 29.1% to 13.7%<br>• Acute diarrhea down from 11.2% to 6.7%

NB: ARIs = Acute Respiratory Tract Infections; HCPs: Healthcare Professionals; PHCs = Primary Healthcare Centers; STIs = Sexually Transmitted Infections; URTIs = Upper Respiratory Tract Infections; UTIs = Urinary Tract Infections

Table S2. - Summary of ASPs and other activities to reduce inappropriate dispensing of antibiotics without a prescription among LMICs and their impact.

Country and reference	Intervention and Aims	Impact of the Intervention
**A) Successful Interventions**		
Bangladesh [98]	• Drug sellers in Dhaka participated in 6 standardized role-playing scenarios<br>• An ARI guideline was developed following the role-play coupled with a a one-day educational course regarding the appropriate management of ARIs<br>• The guidelines only recommended antibiotics for children with complicated ARI	Mixed results:<br>• In children, the dispensing of antibiotics for uncomplicated ARIs decreased (30% baseline vs. 21% post-intervention; p = 0.04)<br>• However, drug sellers were equally likely to dispense antibiotics for complicated ARIs and referrals to physicians for complicated ARIs decreased (70% baseline vs. 58% post-intervention; p = 0.03)<br>• For adults, the dispensing of antibiotics without a prescription remained similar for patients with uncomplicated ARI (48% baseline vs. 40% post-intervention; p = 0.1) but increased among those with complicated ARI (44% baseline vs. 78% post-intervention; p < 0.001)
China [99]	• Multiple initiatives were introduced in Shaanxi Province in China to reduce the purchasing of antibiotics without a prescription given rising concerns<br>• These included:<br>○ Stricter regulations for dispensing antibiotics<br>○ mproved education of pharmacists<br>○ Stipulating there must be a qualified pharmacist's present to dispense antibiotics<br>○ Increased frequency of unannounced pharmacy inspections alongside punishments for abuse	• These multiple measures resulted in:<br>○ Decreased antibiotic sales between 2011 and 2017<br>○ Decreased dispensing of antibiotics without a prescription for a 5 year old child with diarrhoea between 2011 and 2017 - from 72.3% to 50.2% (p<0·0001) among simulated patients<br>○ A reduction in the dispensing of antibiotics for simulated patients with URTIs – down from 95.8% to 69.5% (p<0·0001)
Kenya [83,100-103]	• Multiple activities including:<br>• Education among pharmacists linked to the Pharmacy undergraduate programme at the University of Nairobi (UoN)<br>• Regular interaction with University staff and students regarding key issues surrounding antibiotics and AMR, espeically inappropriate use<br>• The University launched and implemented an AMS curriculum for undergraduate pharmacy students as part of its NAP to reduce AMR	• Low level of dispensing of antibiotics without a prescription (94.1% of antibiotics dispensed with a valid prescription) in the study of Mukokinya et al. (2018) among pharmacies allied to the UoN –<br>• Contrasts with pharmacies not allied to the university and are not superintended by pharmacists - in the study by Muloi et al. (2019), 52% of surveyed pharmacists not allied to the University had sold antibiotics without a prescription<br>• In the study of Opanga et al. (2021) at the start of the COVID-19 pandemic, there was no purchasing of antimicrobials without a prescription among patients with actual or suspected COVID-19 among community pharmacies allied to the UoN. These community pharmacists typically recommended alternative treatments for ARIs

		• This compares with the study of Kimathi et al. (2022) who found 23.4% of patients had self-medicated with antibiotics during the pandemic, 60.6% at the onset of COVID-19 symptoms before confirmatory tests, with 51.5% self-medicating more than once
Namibia [78, 104]	• Education of pharmacists starting in universities and continuing post qualification<br>• Implementation of regulations banning the purchasing of antibiotics without a prescription<br>• Activities of community pharmacies regularly monitored	• In a survey among 100 households in Namibia, typically cold/flu medication, paracetamol, and decongestants were used to treat adults or their children with ARIs including for common colds and influenza. There was no purchasing of antibiotics without a prescription<br>• A similar situation was seen during the COVID-19 pandemic with no change in antibiotic utilization patterns early in the pandemic among 55 community pharmacies surveyed
Republic of Srpska [84,105]	• Multiple interventions including:<br>o Education of pharmacists regarding the appropriate management of infectious diseases including ARIs<br>o Production of guidelines for the 42 most frequent diseases and conditions seen in everyday practice in community pharmacies<br>o Greater enforcement of the regulations of guidelines banning the dispensing of antibiotics without a prescription together with possible fines for violation of laws (Euro500–1500 for pharmacy directors and Euro500–750 for pharmacy technicians)<br>o Special attention given to the importance of adequate communication and skills among community pharmacists especially given their increasing importance	• The dispensing of antibiotics without a prescription decreased from 58% to 18.5% of pharmacies<br>• OTC therapy to alleviate symptoms was offered in 72.3% of pharmacies in 2015 - up from 67.2% in 2010<br>• OTC medication dispensed included analgesics and antihistamines, throat and nasal sprays, decongestants and oral expectorants<br>• Significantly fewer pharmacies dispensed an antibiotic without a prescription where OTC medicines were offered<br>• Encouragingly, the most common reason for not dispensing an antibiotic to simulated clients with ARIs was that antibiotics cannot be dispensed without a prescription
Uganda [106]	• Four-part intervention to improve the management of pediatric febrile illness among drug sellers. These included:<br>o Training and work activities<br>o Provision of information, education alongside communication<br>o Supply activities to drug sellers including diagnostics and medicines<br>o Monthly support supervision via supervisors trained in either pharmacy or clinical medicine	• The intervention increased the appropriate treatment of children with uncomplicated malaria, pneumonia symptoms and non-bloody diarrhoea by 80.2% (95% CI 53.2–107.2), 65.5% (95% CI 51.6–79.4) and 31.4% (95% CI 1.6–61.2) respectively versus the pre-intervention period<br>• Adherence to guidelines during the intervention phase was high without causing excessive prescribing of antimicrobial medicines
**B) Interventions that failed to achieve the desired outcome**		
Chile [107,108]	• Introduction of a law in 2005 in Chile which banned the purchasing of antibiotics without a prescription<br>• Alongside this initial monitoring and enforcement	• The initial enforcement of the law had a modest impact on overall antibiotic sales in the first three years of its implementation (-1.00 DDDs/ 1000 inhabitants per day)<br>• However, a follow-up study five years after implementation found a high number of pharmacies

		(80.3%) were still not complying with the law due to lax monitoring<br>• These findings prompted calls for a greater enforcement of the law to reduce the purchasing of antibiotics without a prescription in the country
Venezuela [107]	• The government implemented policies in an attempt to limit the dispensing of three antibiotic groups without a prescription<br>• However, there were no public awareness campaigns and no real 'enforcement' as this was via government publications with no active campaigns to follow up of the regulations	• As a consequence, there was no decrease in antibiotic utilization levels in the country following the implementation of these modest policies<br>• In fact, the opposite was seen with an actual increase in antibiotic utilisation in the country despite the policies
Vietnam [86]	• There were strict regulations banning the dispensing of antibiotics without a prescription along with the monitoring of pharmacies<br>• However, there were limited sanctions for community pharmacists when dispensing antibiotics without a prescription - a fine of only US$15-25 per documented violation<br>• However, going straight to community pharmacists is seen by patients as typically more convenient and does not waste time to see a physician, which may decrease daily wages<br>• In addition, often limited monitoring of outlets in practice	• 487 out of 1,626 participants taking part in the study involving community pharmacies and drug sellers were able to purchase antibiotics without a prescription<br>• 81.7% were able to purchase antibiotics without a prescription involving 29 different antibiotics<br>• In 86.4% of the situations, participants were prescribed antibiotics by drug sellers<br>Most antibiotics were sold to treat RTIs (61.4%)

Supplementary Table S3: Questionnaire

Antimicrobials dispensing practices from community pharmacies/medical stores during COVID-19 in Punjab

Section 1 Information related to drug sale point

1. Name of division..
2. Name of district..
3. Name of tehsil...
4. Code of pharmacy/medical store...
5. Location of pharmacy/medical store

   Urban
   Rural
6. Presence of pharmacist/pharmacy technicians
   Yes
   No
7. Type of pharmacy/medical store
   Chain
   Independent
8. Number of encounters during study duration..........................

Section 2 Demographic characteristics of the patients/customers

1. Age of patients in years ....
2. Gender

   Male
   Female
3. Category of AM dispensed

   Antibiotics
   Antiviral
   Antifungal
   Anthelmintics
   Antiprotozoal

Section 3

Detail of AM dispended

**Name of agent (Non-proprietary)**	**Indication**	**Route of administration**
Antibiotic		
Antiviral		
Antifungal		
Anthelmintics		
Antiprotozoal		

If antibiotics were dispensed then, detail of antibiotics as per the AWaRe class

1. Access
2. Watch
3. Reserve
